# Texture Classification Using Spectral Entropy of Acoustic Signal Generated by a Human Echolocator

**DOI:** 10.3390/e21100963

**Published:** 2019-10-02

**Authors:** Raja Syamsul Azmir Raja Abdullah, Nur Luqman Saleh, Sharifah Mumtazah Syed Abdul Rahman, Nur Syazmira Zamri, Nur Emileen Abdul Rashid

**Affiliations:** 1 Wireless and Photonic Network Research Centre (WiPNET), Faculty of Engineering, University Putra Malaysia (UPM), Serdang 43400, Malaysia; gs45188@student.upm.edu.my (N.L.S.); s_mumtazah@upm.edu.my (S.M.S.A.R.); nursyazmirazamri@gmail.com (N.S.Z.); 2 Microwave Research Institute, UniversitiTeknologi MARA (UiTM), Shah Alam 40450, Selangor, Malaysia; emileen98@uitm.edu.my

**Keywords:** spectral entropy, acoustic signal, human echolocation, classification, MFCC

## Abstract

Human echolocation is a biological process wherein the human emits a punctuated acoustic signal, and the ear analyzes the echo in order to perceive the surroundings. The peculiar acoustic signal is normally produced by clicking inside the mouth. This paper utilized this unique acoustic signal from a human echolocator as a source of transmitted signal in a synthetic human echolocation technique. Thus, the aim of the paper was to extract information from the echo signal and develop a classification scheme to identify signals reflected from different textures at various distance. The scheme was based on spectral entropy extracted from Mel-scale filtering output in the Mel-frequency cepstrum coefficient of a reflected echo signal. The classification process involved data mining, features extraction, clustering, and classifier validation. The reflected echo signals were obtained via an experimental setup resembling a human echolocation scenario, configured for synthetic data collection. Unlike in typical speech signals, extracted entropy from the formant characteristics was likely not visible for the human mouth-click signals. Instead, multiple peak spectral features derived from the synthesis signal of the mouth-click were assumed as the entropy obtained from the Mel-scale filtering output. To realize the classification process, K-means clustering and K-nearest neighbor processes were employed. Moreover, the impacts of sound propagation toward the extracted spectral entropy used in the classification outcome were also investigated. The outcomes of the classifier performance herein indicated that spectral entropy is essential for human echolocation.

## 1. Introduction

**Figure 1:**
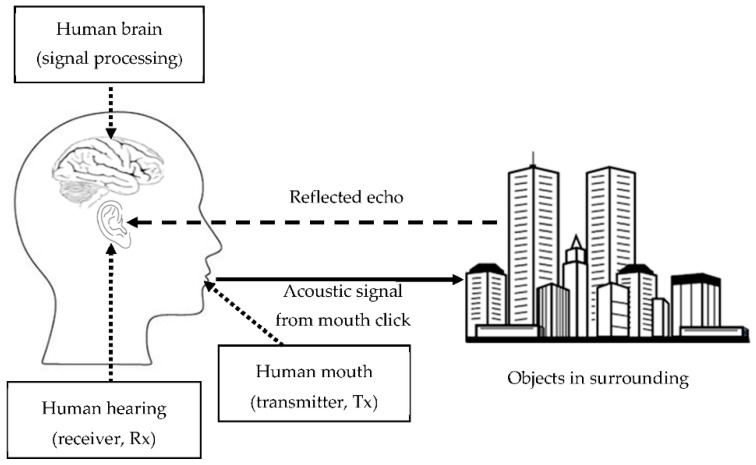
The human echolocation concept.

The phrase “echolocation” was initially used by Griffin to describe bats’ ability to safely navigate and locate their prey using ultrasound call signals [1]. What is less known is that a group of people (often blind people) known as human echolocators have adapted to visualize their surrounding using a similar concept. Human echolocation is the ability to perceive one’s surroundings by listening to the echoes of the active emission of sound signals reflected from an obstacle. Recent studies have reported that these people are able to visualize their surrounding by “seeing through sound”. They exhibit exceptional performance in defining their space, even able to accurately discriminate the profile of objects. This ability has elicited inquiries among scholars on how the space and objects can be recognized using mouth-click signals. Although a series of studies have focused on this question, most works have focused on the perceptual concept rather than technical explanations. It is known that human echolocators depend on their auditory systems in order to translate meaningful cues from mouth-click signals and turn them into visual perception, as illustrated in Figure 1 [1].

It is essential for humans to recognize the acoustic signals entering their ear, mainly for communication and recognition. For comparison, radar and sonar are a good example of man-made sensors which benefit from these classifications; the illumination of a target is associated with detection and classification schemes [2,3,4]. This meaningful information is useful in distinguishing the profile of the detected target, and helps to minimize poor and false detection events. For human echolocation, how people recognize spaces and objects using mouth-click signals has still not been clearly verified. In addition, no studies to date have reported a technical classification process of human mouth-clicks. Moreover, human mouth-clicks do not inherit formant properties like those in typical speech, which have been proven to be strong features in speech recognition. Instead, the multiple frequency components (spectral entropy) found in the signal serve as features for the classification process. This gap should be investigated to assure continuity and to utilize the full potential of human echolocation. We were thus motivated to analyze and design this classification process. 

Studies related to the human auditory system became the primary references in this paper for the classification process of human mouth-clicks. We herein propose a classification scheme for human mouth-clicks using experimental data by utilizing a human auditory model (Mel-frequency cepstral coefficient (MFCC) framework processing). By understanding how human echolocators carry out echolocation (utilizing mouth-click), a new dimension could be opened in the design of man-made sensors (especially for radar and sonar) in the near future. In this paper, we have not made any claim that the classification schemes described closely replicate the strategy used for human echolocation; we present the best intuitive approached for decision-making based on the credible knowledge of human echolocation techniques and the human hearing process.

Hence, this study aimed to investigate the characteristics of the echo signal (acoustic mouth click) reflected from different textures at various distances. We developed a classification scheme to classify textures based on the spectral entropy of the echo signal obtained from Mel-scale filtering outputs (Mel-spectrum), which was incorporated into the MFCC framework. The classification tasks included distinguishing hard, medium, and soft textures, and grouping them into their respective cluster region sources using the reflected echo signal at different distances. The classification routine was realized using K-means process and was validated using K-nearest neighbors (K-NN). The paper is structured as follows: Section 2 provides a description of the characteristics of the human mouth-click signal, and presents a brief explanation of the experimental setup used for data collection, followed by echo signal identification. Section 3 elaborates the flow process of extracting spectral entropy using the K-means approach, followed by texture clustering and classification performance analysis using K-NN. Section 4 evaluates the results and discussion. In chapter 5 provides an outline of the study’s conclusion and the direction of future work.

The study of human echolocators was initiated by Supa et al. nearly half a century ago [5]. They performed experiments with participants who were instructed to tap their heels on the floor in such a way as to create noise for echolocation purposes. A subsequent study conducted by Kellogg found that tongue-clicks, finger-snaps, hissing, and whistling were among signal sources that could be used to echolocate [6]. Rice et al. conducted a subsequent study which revealed that the majority of the participants preferred to use self-generated signals for echolocation [7]. In a subsequent study, Rojas et al. reported that the majority of the participants also used self-generated sounds to echolocate using oral structure [8].

In 2010, Schenkman et al. found that the distance between the target and the human echolocator could reflect echolocation performance [9]. A year later, Schenkman et al. carried out experiments on auditory perception that revealed the importance of pitch and loudness in human echolocation [10]. During the experiments, the participants were asked to listen to sound signals with manipulated pitch and loudness; the results revealed that pitch (spectral) was sufficient to conduct echolocation. In parallel, studies have proven that the information carried via human echolocation processes is adequate to provide the identity of obstacles in a space (the position, size, material, and shape of an object). Rice et al. found the blind participants were able to make a judgment on the effective surface area (size) of the object [11]. Findings from this study found that larger surface areas could reflect much more energy and lead to an improved success rate. A newer study mostly confirmed that humans do echolocate on a daily basis and are able to differentiate shapes and sizes of objects [12]. In separate works, human echolocation has exhibited remarkable performance in discriminating object texture [13,14]. These facts indicate that human echolocation uses multiple cues, which helps them to translate that meaningful information into an accurate result. These important cues include time delay, spectrum (frequency), and amplitude (loudness). The most recent studies assert that spectral entropy plays a major role in determining the effectiveness of human echolocation processes [15]. However, no studies to date have reported a technical analysis of how these people successfully differentiate the shape, size, and texture of objects.

A study revisiting the concept of human echolocation placed an emphasis on the technical analysis perspective. For instance, analysis of the waveform diversity of human mouth-clicks revealed that they are relatively short, wide-band, and have multiple peak frequency components with exceptional resolution detail [16]. Subsequent studies mostly confirmed that mouth-click signals are individually unique, and that their spectral entropy contains multiple frequency components underlying an envelope factor that probably makes up the entire signal [17,18]. Translated into radar and sonar system applications, such properties could resolve detailed Doppler and delay information for accurate detection results.

In addition, speech is undoubtedly human beings’ primary mode of communication. The natural speech communication process involves the use of the mouth to produce an audible signal (acoustic signal), and the use of the ear to interpret this signal [19]. Interestingly, human echolocators also use the mouth–ear mechanism in a secondary sensing modality with which to perceive their surroundings [20]. They listen to the return echo of an active emission acoustic signal generated by a punctuated mouth-click in the space Figure 1. The echo signal is then interpreted by the human brain and translated into meaningful information. Similarly to speech events, human echolocators rely on their hearing sense ability to interpret the reflected echo signals. Thereby, a similar strategy used in speech processing is used in human mouth-click echolocation. Understanding the nature of the biological processes involved is essential to carrying out an analysis of the human mouth-click [21]. For any audible signal, the recognition process in human hearing is enclosed with identical processing manners. It is worth mentioning that the behavior that stands out the most in the human hearing sense is the ability to synthesize an audible signal in a logarithmic scale, in order to accommodate the physical structure of basilar membrane in the inner ear.

Human echolocation studies in recent years have shown exceptional performance and high accuracy while echolocating [20] using the mouth-click signal. Mouth–ear structures have been acknowledged to be used to echolocate, but how they exploit those systems for detection and classification has still not been widely explored. As a result, an initiative to analyze the mouth-click mechanism under the context of human auditory modeling has been introduced, and was exemplified by the successful recognition results for a pair of transmission–echo signals using the Linde–Buzo–Gray vector quantization (LBGVQ) method [22]. The method’s principle is to extract cepstral entropy between transmission–echo signals using Mel-frequency cepstral coefficient (MFCC) processes. As a result, features of a true pair of transmission–echo signals are scattered within the same cluster. Improved detection of human mouth-clicks was achieved with a bio-inspired (BI) processing approached over the matched filter (MF) outcome [23,24]. The BI process utilized a gammatone filter (GF) process in order to synthesize the mouth-click that was used in the detection process. In addition, the synthesized mouth-click signal using GF was extended into ambiguity function (AF) analysis [25]. Results of the analysis revealed that the ability to resolve Doppler-delay information from each filter output was unique. Thus, it became worthwhile to study the human mouth-click from a human modeling perspective, as the signal source was a human being.

## 2. Signal Characteristics and Experimental Setup

The human mouth–ear communication framework can be replicated by a speaker–microphone setup. The transmission source of the mouth-click signal that was used throughout the data collection is publicly available and can be retrieved from reference [20]. In this section, the characteristics of the mouth-click used for artificial data collection are described in brief. We acknowledge that there are blind people who use finger-snaps, a tapping cane, and hand claps to help them navigate safely. However, this paper only considered signals from mouth-click sounds, as (i) recent scientific studies have reported that human echolocators often use sound from mouth-clicks for echolocation processes. Several studies have emphasized a technical perspective of this method [16,17,18,20], and (ii) the signal has been properly recorded and made publicly available to be used for research [20].

### 2.1. Human Mouth-Click Signal Characteristics

**Figure 2:**
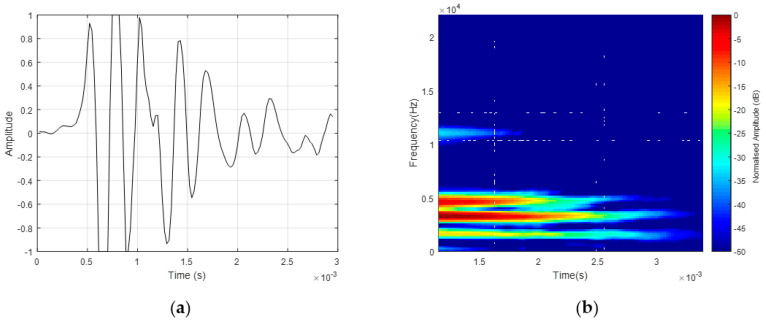
Characteristics of the human echolocator’s mouth-click: (**a**) time-domain signal and (**b**) spectrogram [2].

A single human echolocator signal was employed in this study, belonging to a blind person with reportedly exceptional echolocating skill. Moreover, as young as 13 months old, this person was diagnosed with the retinoblastoma that caused his blindness, and since then has utilized echolocation in his daily activities. The recorded mouth-click was digitized using a sampling frequency, *f_s_*, of 44,100 kHz in an uncompressed Windows audio format (wav). This was able to retain the spectral entropy of the mouth-click in a digital format based on the Nyquist theorem. The duration of the mouth-click was relatively short, approximately 3 ms, without any specific modulation scheme, as shown in Figure 2a. The spectrogram analysis shown in Figure 2b revealed an entropy identity with the presence of multiple frequency components in two separate regions, namely the main frequency region and upper frequency region. The major energy could be found in the main frequency region.

Despite having unique waveform diversity, these multiple frequency components are likely to cause undesirable output when performing the detection process using MF, due to the abundance of multiple local maxima and low sidelobes level (SLL). This is an undesirable outcome especially in detection outputs, where the system is prone to poor detection and false alarms. To tackle the issue, an alternative approach using BI entropy has been reportedly used in recent analyses [22,24]. More specifically, the BI-incorporated GF process first synthesizes the signal, then performs a multi-stage correlation process, followed by summing all correlation products to improve the detection results. Thus, it worthwhile to explore the potential of classification performance by utilizing extracted spectral entropybased on a human auditory modeling approach, as presented in this paper. Details of entropy extraction using the MFCC framework are discussed briefly in Section 3.

### 2.2. Human Mouth–Ear Experimental Setup

**Figure 3:**
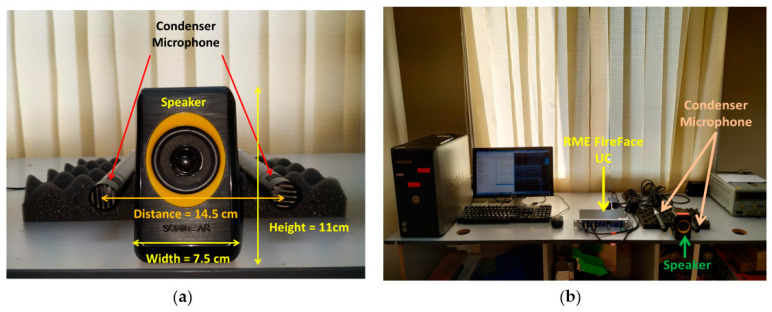
Experiment setup for an artificial human echolocator: (**a**) speaker–microphone layout and (**b**) complete layout.

Figure 3a shows the configuration of the human mouth–ear system modeled with a speaker–microphone setup. A condenser microphone was chosen as it offered improved input sensitivity, lower noise, and the widest frequency response of the dynamic microphones. Both the speaker output and the microphone input connector were relayed into RME Fireface UC, acting as DAC and ADC and were linked to the computer via high-speed USB 2.0, as shown in Figure 3b. Data collection throughout the experiment was controlled using Matlab for flexible data manipulation in the classification tasks.

**Figure 4:**
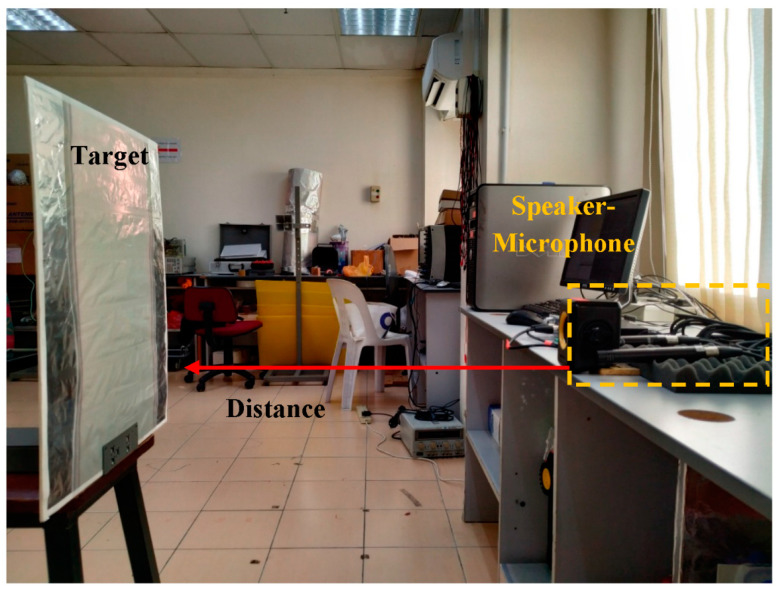
Target distance from the speaker–microphone setup.

The target was placed on top of a flat wooden chair in line of sight and facing the speaker–microphone layout to help maximize the reflection properties of the individual target with different textures. The target distance was measured from the baseline of the speaker–microphone, at 50 and 100 cm, as illustrated in Figure 4. Such distance values were reasonable and correspond to the average human step and stride length at a normal walking pace, which is reportedly 70 cm on average [26]. Considering a range of scenarios, 50 and 100 cm should be appropriate to represent actual human echolocation. Throughout the data collection, the air-conditioner temperature was set to 16 °C in order to maintain the surrounding ambient temperature and humidity.

**Figure 5:**
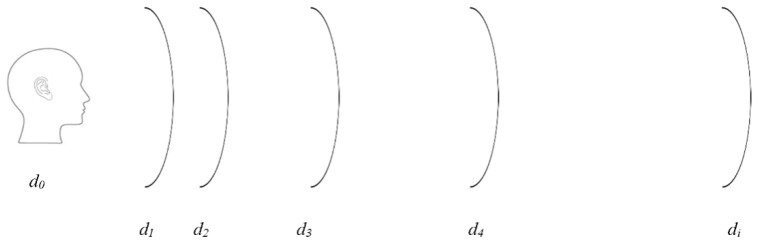
Sound pressure level law theory.

Accordingly, it was essential for each texture to have an identical effective surface area in order to standardize the effect of sound pressure level (SPL) and sound intensity (SI) upon the collected echo signals [27,28]. Likewise, both SPL and SI were associated with the distance the sound traveled. Importantly, as sound radiates freely in space, it is bound to experience a decrease in sound pressure *p* (N/m^2^), as illustrated in Figure 5. Thus, *p* is inversely proportional with distance *d*, as shown in Equation (1). Consequently, the relation between SPL and *d* can be described using the law of sound pressure, *ΔP_p_*, as expressed in Equation (2)**,** where *D_r_* is the distance ratio corresponding to the sound origin and *d_o_* is sound source location. In the experiments herein, understanding of this phenomenon was crucial, as a microphone records data in a similar manner to SPL.

(1) $$p\;∼\frac{1}{d}$$

(2) $$\Delta P_{p}=20\;log\left(D_{r}\right);\;D_{r}=\left(\frac{d_{0}}{d_{i}}\right)\;i=1,2,3,\ldots ,N$$

**Figure 6:**
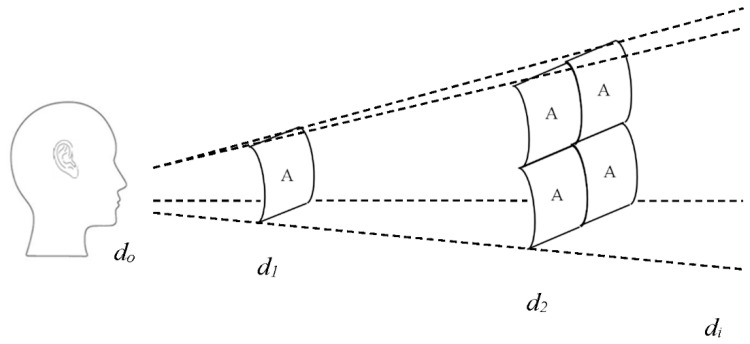
Sound intensity (SI) associated with the inverse square law theory.

In addition, SI significantly decreases linearly with an increase in distance from the sound origin when traveling in free space. Degradation of SI value occurred because the sound energy continued to spread across a much larger surface area as it traveled, as illustrated in Figure 6. Thereby, for any given distance that doubled that from the sound source, the intensity I (W/m^2^) was bound to decrease by a quarter of its original value following the inverse square law. The SI was calculated using Equation (3), with P_ac_ (Watt) being the acoustic power at *d_o_*, A (m^2^) the sphere area, and d (m) the distance.

(3) $$I\;∼\frac{1}{d^{2}};\;I=\frac{P_{ac}}{A};\;A=4\pi \times r^{2}$$

The target base was made of polyvinyl chloride (PVC) and wrapped with different materials found from the hardware shop, as detailed in Table 1. A total of three materials were used for distinguishing texture, namely soft, medium, and hard materials, with a surface area of 3025 cm^2^. Each material has distinct composition characteristics; thus, the absorption properties varied accordingly, as discussed in Section 3.2 [29]. As this was a preliminary investigation, we scaled down the scope of this paper to classifying three different textures at the respective distances.

The methodology discussed above emphasized the introduction of diverse elements relative to reflected echo signals so as to classify their behavior and performance, which was a major aim of the analysis in this paper.

### 2.3. Signature of Reflected Echo Signal

The spectral entropy in the sound signal was associated with sound propagation effects while it traveled the distance described in Section 2.2. Thus, these two factors were expected to influence the reflected echo signal quality received by the microphone. In order to analyze the texture classification at a certain distance, the correct reflected signal had to be identified from the raw data. The true echo signal was extracted via Equation (4), with *d* as the distance between the target and the speaker–microphone, *t* as the returned time echo signal into the microphone, *θ* as the elevation of target with respect to the speaker–microphone, and *C* as the speed of sound in air, which equates to 342 m/s,

(4) $$d=\frac{C\cdot t}{2}\cos\theta .$$

**Figure 7:**
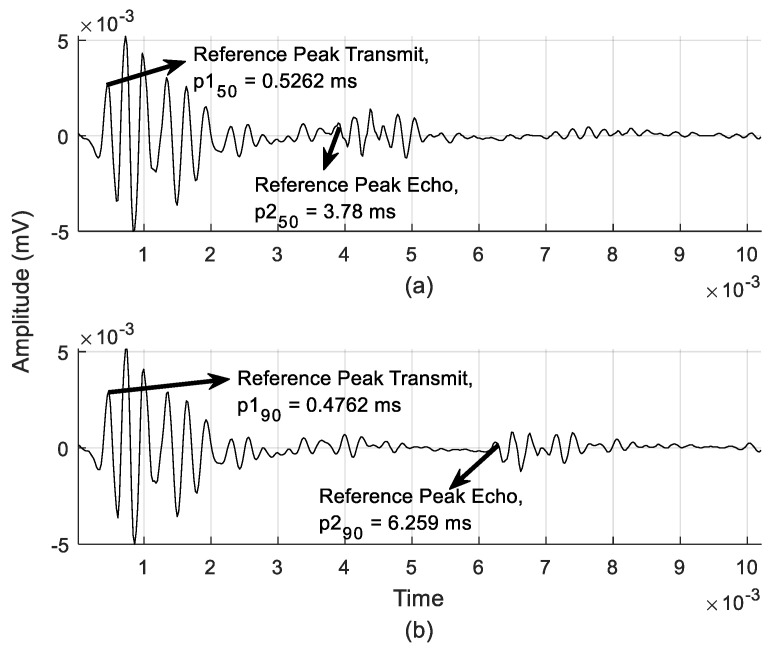
Experimental raw data containing transmit leak and echo signal: distance of (**a**) 50 cm and (**b**) 100 cm.

Moreover, using Equation (4), the echo signal from the raw data could be estimated and extracted. Figure 7a shows the experimental data for the 50 cm distance target given *t* = 3.2538 ms, resulting in *d* = 55.64 cm. For the distance target at 100 cm and *t* = 5.7828 ms, *d* was 98.89 cm, as shown in Figure 7b. The offset delay for the reflected echo signal from its actual calculation in Equation (4) was caused by atmospheric factors, which can significantly influence the speed of sound [30].

**Figure 8:**
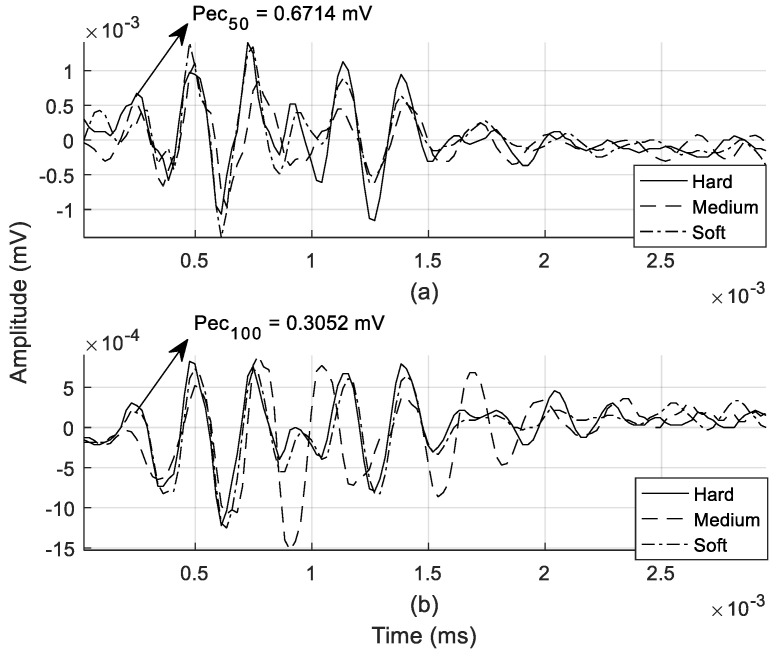
Reflected echo signal for all textures: distance of (**a**) 50 cm and (**b**) 100 cm.

The SPL effect, as discussed in Section 2.2, was discernible from the extracted echo signal; differences in the amplitude are highlighted in Figure 8. At any given point of the target from the speaker–microphone, the echo signal experienced an attenuation effect, as demonstrated in the amplitude.

**Figure 9:**
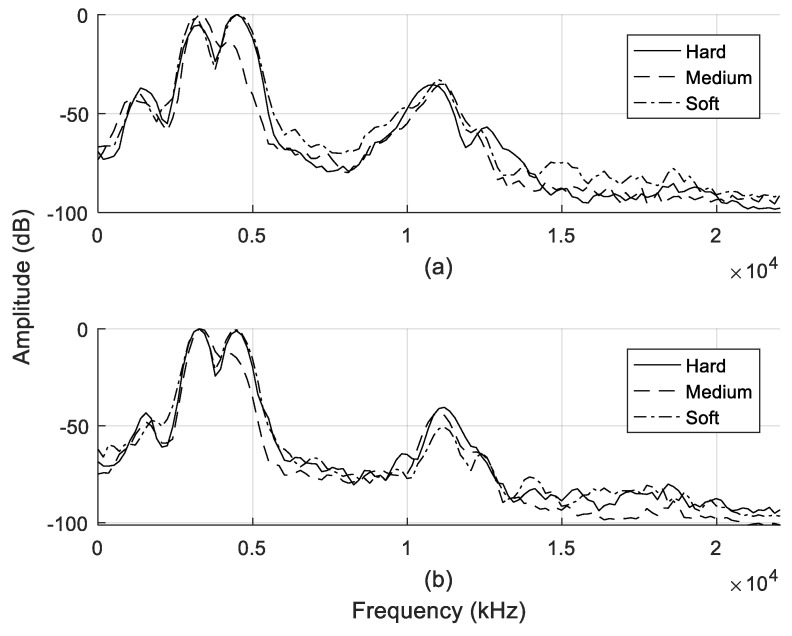
Power spectrum density (PSD) of reflected echo signals: distance of (**a**) 50 cm and (**b**) 100 cm.

The time-domain characteristic of a sound signal provides a first glance at the spectral entropy of the energy profile of the individual echo signal. This energy (W/m^2^) denotes the SI in Equation (3). The visible variation in normalized amplitude between echoes at 50 and 100 cm was due to sound energy being dissipated across a larger surface area as it traveled from the sound origin, as shown in Figure 9.

## 3. Spectral Entropy Features and Classification Framework

In classification tasks, it is essential to obtain a set of features that helps to translate meaningful information accurately to discriminate the respective identities of the features [2,31,32]. In human speech recognition applications, extracted formant coefficients are often harvested and manipulated and then used as features [33,34,35,36]. Speech recognition processes in the present-day scenarios have reached a level of maturity where they are able to produce exceptional performance. Consequently, speech recognition processing schemes utilizing spectral entropy have been exploited in non-speech signal applications, e.g., smart devices [37,38], robotics [39], and surveillance cameras [40].

To accomplish the features extraction task in this paper, a Mel-scale filtering process (incorporated into the MFCC framework) was deployed in the classification campaign. The feasibility of MFCC to contribute to obtaining exceptional results in speech recognition applications has already been clearly demonstrated [37]. In a wider sense, if an extracted entropy from a Mel-scale filtering process is able to classify these textures which are similar in surface area (2D shape), then it could be much easier for this method to segregate a target with a complex curved shape and materials (3D in shape) for subsequent analysis. The motivation to use spectral entropy for features arose because the mouth-click signal was made up of multiple frequencies, as described in Section 2.1.

### 3.1. MFCC Structure

**Figure 10:**
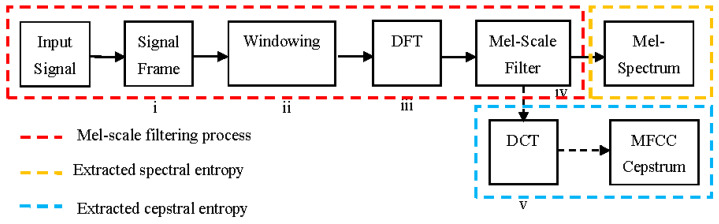
Block diagram for features extraction using the Mel-frequency cepstral coefficient (MFCC).

The conventional of the MFCC framework consists of five major sub-processes of the method, namely, (i) signal framing, (ii) windowing, (iii) discrete Fourier transform (DFT), (iv) Mel-scale filtering and smoothing, and (v) discrete cosine transform (DCT), as graphically illustrated in Figure 10. To avoid conflicts of interest, our aim was to use extracted spectral entropy (Mel-spectrum) from the Mel-scale filtering process for classification campaign (see Section 3.2). Hence, in this section we have described the complete MFCC framework for easier knowledge on obtaining spectral entropy via Mel-scale filtering process (since the Mel-scale filter is embedded in the MFCC framework).

The extracted entropy process was initiated via the breakdown of the mouth-click echo signal into a frame corresponding to a number of Mel-scale filters, *K*. Here, *K* was defined to be equal to 40, which is sufficient to represent the echo spectral amplitude in a minimum windowing size of approximately 15.87 ms after zero-padding (as the length of mouth-click itself is about 3 ms). Next, using a Hammering filter, the windowing process minimized the effect of sharp edges.

**Figure 11:**
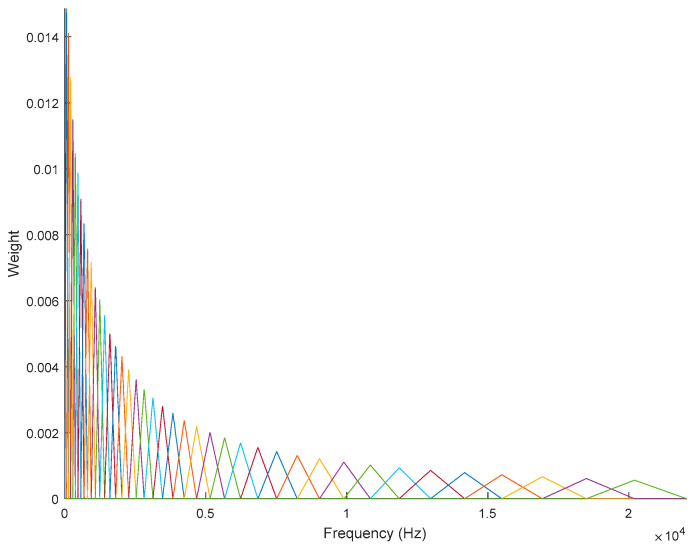
Mel-scale filter-bank with 40 channels.

Subsequently, DFT was taken on each frame to obtain the spectral amplitude (frequency domain), after which such amplitude was passed onto the Mel-scale filter-bank *m*, where *M_n_*(*f*)** is an inverse representation of *m*, as expressed in Equation (5). The Mel-scale filter-bank response corresponding to Equation (5) is illustrated in Figure 11.

Mel-filtering aims to make spectral content smooth, in preparation for meaningful features representation. In addition, Mel-scale filtering processes helped to exhibit spectral content, mimicking the human auditory system using the weight Mel-scale filter-bank, *W_k_*(*f*)** given in Equation (6), where *X*(*f*)** is DFT bin energy. With this scheme, it was expected that a robust and efficient task for a specific application could be obtained.

(5) $$m=2595\log_{10}\left(1+\frac{f}{700}\right),\;M_{n}\left(f\right)=700\left(10^{m/2595}-1\right),\;n=1,\;2,\;3,\;\ldots ,\;K.$$

(6) $$W_{k}\left(f\right)=\sum_{f}M_{n}\left(f\right)\cdot {\left|X\left(f\right)\right|}^{2}.$$

As mentioned earlier in this section, we used the Mel-scale filtering process to extract features for classification tasks. Hence, the features extraction process via the MFCC framework used in this paper was limited by the Mel-filtering process (see Figure 10). In effect, to obtain cepstrum entropy from the Mel-scale filtering output, it had to be converted into a time-domain representation using DCT termed as the MFCC coefficient *C_k_*(also known as cepstrum entropy). Thus, *C_k_* process could be expressed via Equation (7) and could be used as classification features in future works.

(7) $$C_{k}=\overset{K}{\underset{k=1}{\sum }}W_{k}\left(f\right)\cos\left[n\left(k-\frac{1}{2}\right)\frac{\pi }{K}\right]$$

### 3.2. Mel-Spectrum Output

**Figure 12:**
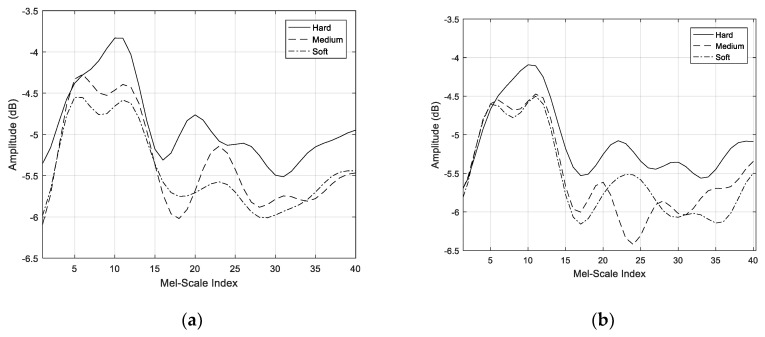
Mel-spectrum output: distance of (**a**) 50 cm and (**b**) 100 cm.

Each of the coefficients extracted during the MFCC process can be mined for meaningful features input depending on the specific tasks. Our approach employed the Mel-scale filtering process as a baseline method to extract features of the mouth-click signal, because it provided an effective indiscriminate texture cluster at respective distances. As such, the Mel-scale filter-bank output in Equation (6) was exploited as features for the classification campaign, due to its strength in presenting the spectral entropy of the mouth-click signal. The Mel-spectrum output of each texture at the distances of 50 and 100 cm are shown in Figure 12a,b respectively. The Mel-spectrum output was able to resolve the spectral features because the human auditory system was able to perceive the loudness in an approximately logarithmic manner. Compared to the PSD results in Figure 9, the Mel-spectrum output managed to discriminate the spectral entropy of individual textures with much more detailed representation.

### 3.3. Classification Assignment Using K-Means and K-NN Validation

**Figure 13:**
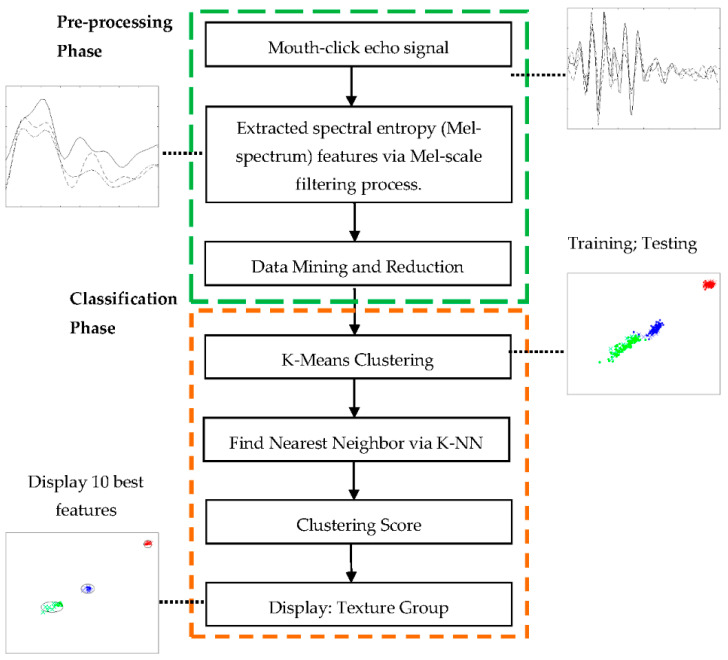
Classification block diagram used to distinguish different textures from mouth-click.

Figure 13 displays the block diagram used for the classification process in this paper. It was divided into two sub-processes: a signal pre-process that synthesized the mouth-click echo using the Mel-scale filtering process to obtain the Mel-spectrum output as features input, and a classification task realization process using K-means to distinguish the cluster of each texture. Next, the information containing the clustering vector from the K-means process was handed over to the K-NN process for performance validation. A built-in function was carried out in Matlab in this study to accommodate the K-means and K-NN tasks.

Under the machine learning philosophy, features extraction, data mining, and data simplification are welcome steps in achieving the desired outcome prior to a classification campaign [41,42,43,44] For this objective, the authors of this study created a database using the calculated mean values of the extracted spectral entropies and arranged them into a matrix, after which they were tagged according to their texture. Once this was completed, relaying into the classification phase could follow using the K-means process. Essentially, K-means group a large number of data samples into a specific number of clusters. To achieve this task, K-means minimize the total intra-cluster variance, represented in Equation (8) as *J*, where *a* indicates a number of clusters, *b* is the number of cases, *x_k_* is cases corresponding to *b*, *c_j_* is a clustering of *a*, and *D_j_* is the distance function. Next, the K-NN process calculated the performance between the training and testing of each cluster texture, which can be generally expressed in Equation (9) as *K_N_*. Moreover, the consistency of each cluster was interpreting using silhouette value. Finally, the clustering performance was further validated with a confusion matrix, and the cluster results were later displayed.

(8) $$J=\overset{a}{\underset{j=1}{\sum }}\overset{b}{\underset{k=1}{\sum }}∥{x_{k}}^{j}-{c_{j}∥}^{2},\;D_{j}={x_{k}}^{j}-c_{j},$$

(9) $$K_{N}=\;\sqrt{\overset{m}{\underset{j=1}{\sum }}D^{2}}$$

A total of 80 and 20 datasets from each texture were used for the training and testing tasks at distances of 50 and 100 cm, respectively. Left and right signal data from the microphone were equally employed for the 80 training data samples, and 20 for the test data sample tabulated in Table 2. To ensure that the 80 datasets used for training were adequate for the features generalization stage, we conducted a consistency test using analysis of variance (ANOVA). The ANOVA analysis measured the variation between columns (signal) in dataset, then calculated the probability of the test statistic, *p_t_* from the F-statistic table denoted as *P*(*F > p_t_*)**. Larger *p_t_* value indicates that the differences between the columns tested were less significant, and vice versa for smaller values. Based on our results, the individual signal of each dataset score over 90% (translated into a percentage) from the ANOVA analysis is tabulated in Table 2. Moreover, it indicates that the signals used to create the dataset for classification were less significant (consistent signal). Hence, we can conclude that the total of 80 datasets used for data training was adequate in this paper.

## 4. Results Evaluation and Discussion

### 4.1. Clustering Results

**Figure 14:**
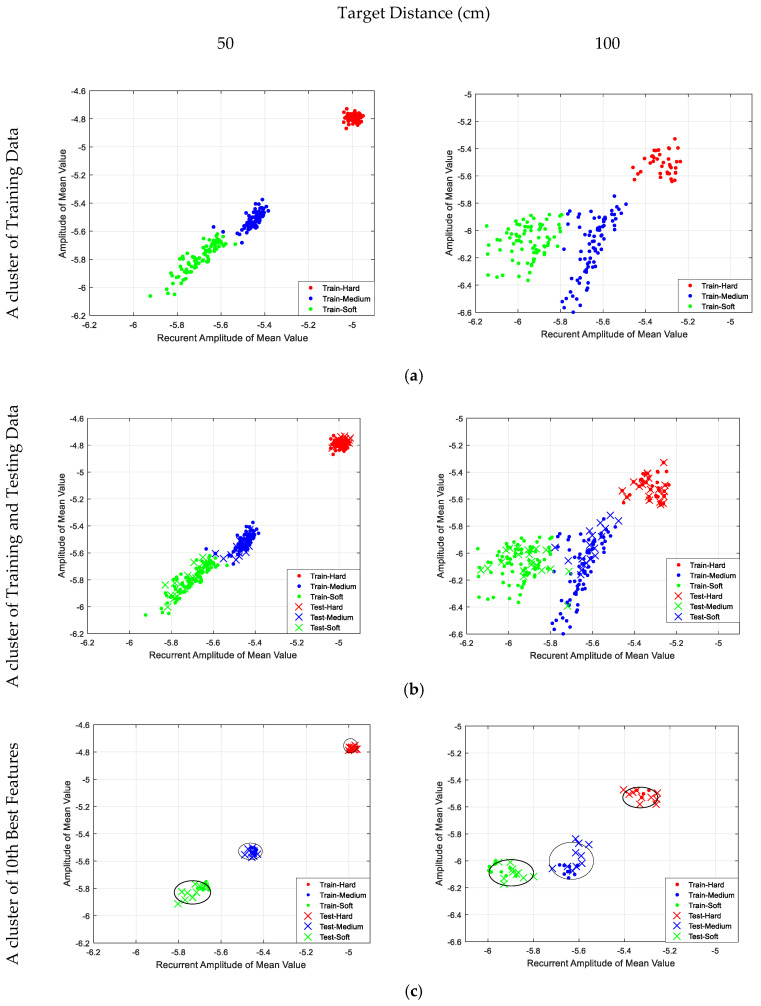
Clustering region of all textures: (**a**) cluster of training data; (**b**) cluster of training and test data; (**c**) cluster of 10 best features.

The clustering region for the training data using K-means environment revealed a visible separation region between the textures, as shown in Figure 14a. Similarly, a clustering texture region for the test data plotted on top of the training data appeared, as shown in Figure 14b. Figure 14b revealed that there was a testing coefficient for medium and soft textures breaching the respective clusters at both distances. There is a high possibility that this event occurred due to ambient noise recorded along with data collection, which was reflected during the K-means process (generalization stage). The features output obtained from the K-means process was used to determine the nearest neighbor features via K-NN process. To further improve the classification performance, we decide to perform dimension reduction by choosing only ten of the nearest neighbor features between the training and testing data from each cluster for the verdict, as shown in Figure 14c.

### 4.2. Silhouette Results

**Figure 15:**
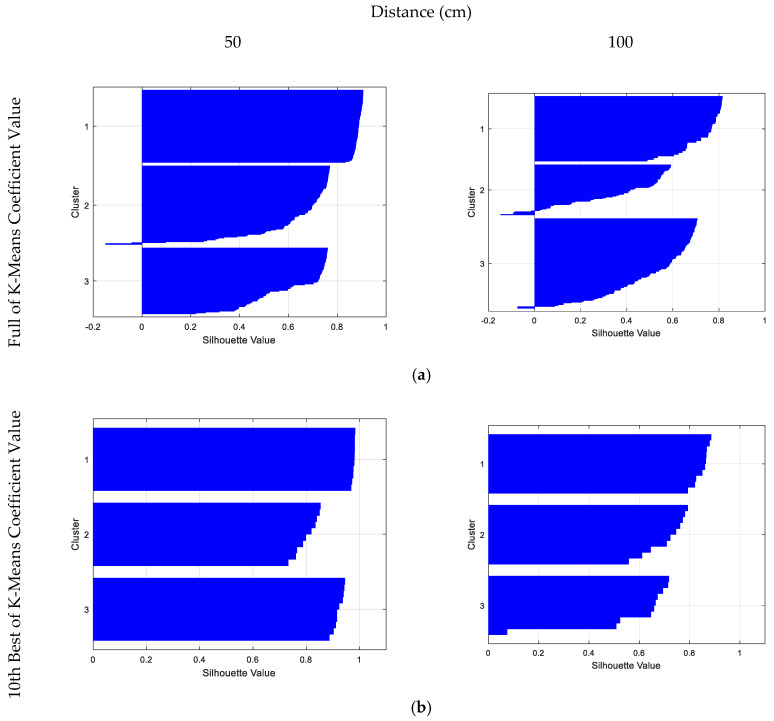
Silhouette plot of different textures (cluster 1 for hard, cluster 2 for medium, and cluster 3 for soft) at different distances; (**a**) full set of K-means coefficient values and (**b**) 10 best K-means coefficient values.

**Figure 16:**
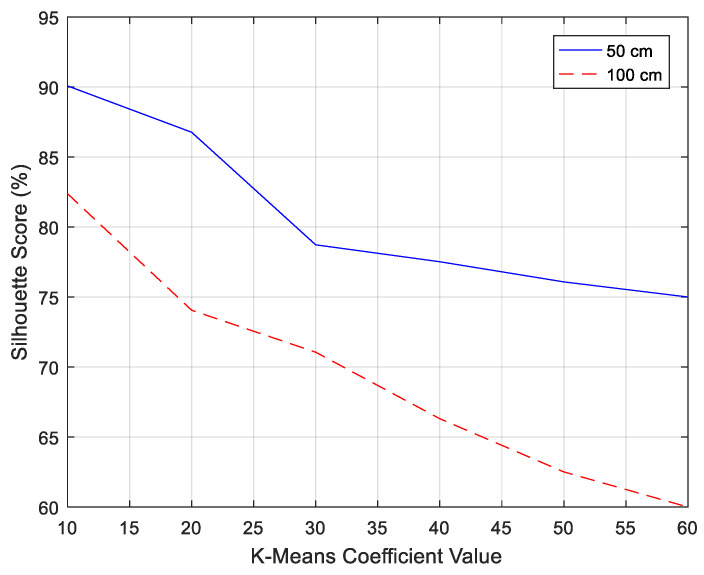
Silhouette score trending.

The clustering behavior shown in Figure 14b,c was monitored using a silhouette function in Matlab, which took over the vector corresponding to the coordinates, points, and column from the K-means process. It measured the degree of similarity between points within a cluster, defined as *Cl_i_*, as given by Equation (10)**,** where *a_i_* is the initial point and *b_i_* the next iteration point. The silhouette distribution pattern for the full K-means coefficient value was better at the distance of 50 cm compared to at the distance of 100 cm, with the cluster distances receiving silhouette plot scores of 75% and 60%, respectively, as shown in Figure 15a. For comparison, Figure 15b shows that dimension reduction with the 10 best K-means coefficient values significantly superseded the silhouette value at both distances, 50 cm and 100 cm, with scores 90% and 82.38%, respectively, against the values shown in Figure 15a. We manage to optimize the classification score and concluded by using the 10 best K-means coefficient values to improve the classification performance, as presented in Figure 16.

(10) $${\mathrm{Cl}}_{i}=\;\frac{\left(b_{i}-a_{i}\right)}{\max\left(a_{i},b_{i}\right)}$$

### 4.3. Confusion Matrix

The final texture classification results corresponding to Figure 14c are tabulated in Table 3, revealing improved performances with 100% scores at the distances of 50 and 100 cm. It is worth noting that the dimension reduction significantly improved the classification of human mouth-clicks by selecting the strong features which reflected good performance. Moreover, three significant findings from the results are worthy of being stressed: (i) SPL and SI may be the factors that reflect the spectral entropy of the reflected echo signals, (ii) dimensional reduction helped to significantly improve the classification performance by selecting the strongest features from the K-means coefficient values, and (iii) these factors helped to build a strong perception of obstacle texture using an echolocation process. These pieces of information could be very useful for blind people, as well as for building an artificial system able to echolocate, which could be a promising direction for future man-made sensor applications. Furthermore, the results suggested that the MFCC framework could become a suitable process used to exploit the spectral entropy of human mouth-clicks, as all textures and both distances (50 and 100 cm) scored full marks (100%), as shown in Table 3.

## 5. Conclusions

This paper presented classification results for human mouth-clicks, the motivations for which sprouted from classifying speech signal processes by means of the MFCC framework for spectral entropy extraction. Artificial data of human mouth-clicks were collected using off-the-shelf audio devices that have been described comprehensively herein. Moreover, the frameworks of the classification framework have been explained in detail, from top to bottom of the processing scheme. The extracted entropy of Mel-spectrum output as a feature vector yielded exceptional outcomes. The experimental result and analysis justified the combination of MFCC framework, K-means, and K-NN as a viable model for classifying human mouth-clicks. It is also worth noting that the distance significantly reflected clustering outcomes, with a shorter distance producing better silhouette values subject to sound propagation.

Overall, the spectral entropy from Mel-spectrum output provided sufficient features for the classification task discussed in this paper. This study was the first to conduct an analysis regarding the classification of human mouth-clicks utilizing Mel-spectrum output as a source modality for features. In the long run, several improvements need to be considered to address gaps in this paper. At this stage, it seems that there are plenty of strategies to be learned from the classification of texture using mouth-clicks. However, we also need to be realistic about the applications of the analysis discussed in this paper. The performance of human mouth-click classification could be benchmarked using different human auditory model processes for spectral entropy extraction, in order to find the model that is best fitted for actual human echolocation applications. Diverse spectral entropies using various object profiles (e.g., size and stiffness) should be created in order to evaluate the effectiveness of spectral entropy of mouth-clicks in classification of various objects. These recommendations are just a few factors that will help to ensure the continuing study of human mouth-clicks, especially for classification tasks. As such, they can be exploited by others in relevant fields as be fits the nature of their studies. Furthermore, it such studies will help to validate the credibility of the knowledge discussed in this paper. Moreover, the findings discussed in this paper can contribute towards realizing the development of human echolocator aid devices, and could benefit human echolocators who are learning and polishing their skill in order to achieve accurate results. In addition, the technique can be applied to radar and sonar systems with appropriate frequencies.

## Figures and Tables

**Table 1:** Description of material used to wrap polyvinyl chloride (PVC) board.

Texture	Material	Dimension (cm)	Images
Hard	Flat PVC board wrapped with aluminum foil	L = 50 H = 50 W = 0.5	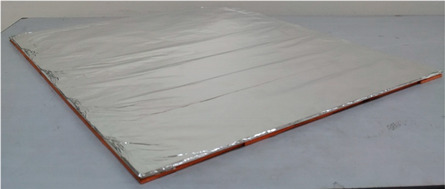
Medium	Rubber mat	L = 50 H = 50 W = 0.8	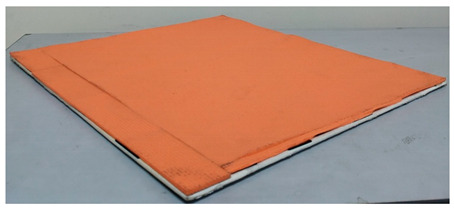
Soft	Sponge	L = 50 H = 50 W = 4	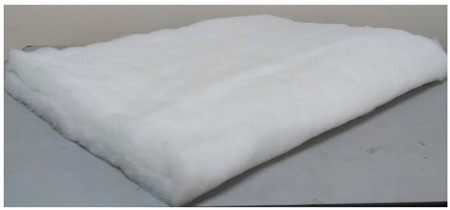

**Table 2:** Summary of datasets used in the classification process.

Distance	Texture	Dataset of Signal (Recorded by Microphone)				
		Training		ANOVA Probability	Testing	
		Left	Right		Left	Right
**50 cm**	Hard	40	40	0.9990	10	10
	Medium	40	40	0.9810	10	10
	Soft	40	40	0.9850	10	10
**100 cm**	Hard	40	40	0.9266	10	10
	Medium	40	40	0.9031	10	10
	Soft	40	40	0.9132	10	10

**Table 3:** Confusion matrix for classification performance.

Distance (cm)	Texture	Confusion Matrix Score (%)		
		Hard	Medium	Score
**50 cm**	Hard	100	0	0
	Medium	0	100	0
	Soft	0	0	100
**100 cm**	Hard	100	0	0
	Medium	0	100	0
	Soft	0	0	100
